# BCL2A1 and CCL18 Are Predictive Biomarkers of Cisplatin Chemotherapy and Immunotherapy in Colon Cancer Patients

**DOI:** 10.3389/fcell.2021.799278

**Published:** 2022-02-21

**Authors:** Taohua Yue, Xiangzheng Liu, Shuai Zuo, Jing Zhu, Jichang Li, Yucun Liu, Shanwen Chen, Pengyuan Wang

**Affiliations:** ^1^ Division of General Surgery, Peking University First Hospital, Peking University, Beijing, China; ^2^ Department of Thoracic Surgery, Peking University First Hospital, Peking University, Beijing, China

**Keywords:** chemotherapy, immunotherapy, BCL2A1, CCL18, PD-L1, colon cancer

## Abstract

**Background:** Cisplatin enhances the antitumor T cell response, and the combination of PD-L1 blockade produces a synergistic therapeutic effect. However, the clinical correlation between cisplatin and immunotherapy in colon cancer (CC) is unknown.

**Methods:** Using the “pRRophetic” package, we calculated the IC50 of cisplatin. The correlation between cisplatin IC50, cisplatin resistance–related genes (CCL18 and BCL2A1), and immunotherapy were preliminarily verified in TCGA and further validated in independent cohorts (GSE39582 and GSE17538), cisplatin-resistant CC cell line DLD1, and our own clinical specimens. Classification performance was evaluated using the AUC value of the ROC curve. Scores of immune signatures, autophagy, ferroptosis, and stemness were quantified using the ssGSEA algorithm.

**Results:** Based on respective medians of three CC cohorts, patients were divided into high- and low-IC50 groups. Compared with the high IC50 group, the low-IC50 group had significantly higher tumor microenvironment (TME) scores and lower tumor purity. Most co-signaling molecules were upregulated in low IC50 group. CC patients with good immunotherapy efficacy (MSI, dMMR, and more TMB) were more attributable to the low-IC50 group. Among seven shared differentially expressed cisplatin resistance–related genes, CCL18 and BCL2A1 had the best predictive efficacy of the above immunotherapy biomarkers. For wet experimental verification, compared with cisplatin-resistant DLD1, similar to PD-L1, CCL18 and BCL2A1 were significantly upregulated in wild-type DLD1. In our own CC tissues, the mRNA expression of CCL18, BCL2A1, and PD-L1 in dMMR were significantly increased. The high group of CCL18 or BCL2A1 had a higher proportion of MSI, dMMR, and more TMB. IC50, CCL18, BCL2A1, and PD-L1 were closely related to scores of immune-related pathways, immune signatures, autophagy, ferroptosis, and stemness. The microRNA shared by BCL2A1 and PD-L1, hsa-miR-137, were significantly associated with CCL18, BCL2A1, and PD-L1, and downregulated in low-IC50 group. The activity of the TOLL-like receptor signaling pathway affected the sensitivity of CC patients to cisplatin and immunotherapy. For subtype analysis, immune C2, immune C6, HM-indel, HM-SNV, C18, and C20 were equally sensitive to cisplatin chemotherapy and immunotherapy.

**Conclusions:** CC patients sensitive to cisplatin chemotherapy were also sensitive to immunotherapy. CCL18 and BCL2A1 were novel biomarkers for cisplatin and immunotherapy.

## Introduction

According to Global Cancer Statistics 2020, colon cancer (CC) is the second most common cause of cancer-related deaths worldwide, whose incidence rate ranks third (Sung et al., 2021). From the histological classification, it is mainly colon adenocarcinoma (COAD), which accounts for about three quarters. It can be seen that CC is a global health problem that needs to be solved urgently.

In addition to surgery, CC can be treated with radiotherapy and chemotherapy, molecular targeted therapy, and emerging immunotherapy (Dekker et al., 2019; Johdi and Sukor, 2020). Immunotherapy refers to a treatment method that artificially enhances or suppresses the immune function of the body to achieve the purpose of curing diseases. Previous studies show that biomarkers for immunotherapy include tumor microenvironment (TME) scores (Xia et al., 2021), tumor infiltrating immune cell (TIIC) abundance (Zeng et al., 2020), expression of immune-related genes (co-inhibitory molecules (T cell and APC cell), Type I and II IFN response molecules, co-stimulatory molecules (T cell and APC cell), cytolytic activity molecules) (Liu X et al., 2021), microsatellite stability (MSI), deficient mismatch repair (dMMR), tumor mutational burden (TMB) (Halama et al., 2016; Duffy and Crown, 2019), immune-related signatures, pathways, somatic mutation frequency (Peng et al., 2016), and activity of autophagy (Ramakrishnan et al., 2012; Sirichanchuen et al., 2012), ferroptosis (Kim et al., 2018; Wang W et al., 2019), and stemness (Hu et al., 2019; Unver, 2021).

Previous research proves that cisplatin augments antitumor T cell responses, leading to a potent therapeutic effect in combination with PD-L1 blockade (Luo et al., 2019; Wakita et al., 2019). Then, we studied the connection between cisplatin chemotherapy and immunotherapy in CC and tried to reveal which CC patients were suitable for cisplatin chemotherapy and immunotherapy and molecular characteristics.

In our study, based on the “pRRophetic” package and transcriptome data, we predicted the half maximal inhibitory concentration (IC50) of cisplatin in CC patients (TCGA, GSE39582, and GSE17538). Based on the biomarkers for immunotherapy, we concluded that the low-IC50 group of CC patients might benefit more from immunotherapy. Besides this, the cisplatin resistance–related genes CCL18 and BCL2A1 could predict the sensitivity of cisplatin chemotherapy and immunotherapy in CC patients, simultaneously. For wet experimental verification, in wild-type and cisplatin-resistant DLD1 cell lines verified by CCK8 experiments, similar to PD-L1, CCL18 and BCL2A1 could predict the efficacy of cisplatin chemotherapy. In the CC specimens of our hospital, compared with pMMR tissues, the mRNA expression of CCL18, BCL2A1, and PD-L1 in dMMR were significantly increased.

Previous studies show that PD-L1 and chemotherapy resistance interact with each other in the biological and functional cascade through microRNA regulation (Xu et al., 2016). On the ENCORI website, we extracted shared miRNAs of PD-L1, CCL18, and BCL2A1. Results showed that hsa-miR-137 was the most potentially predictive miRNA for cisplatin chemotherapy and immunotherapy.

Both functional enrichment analysis (GO-MF and KEGG) and gene set enrichment analysis (GSEA) were applied for the identification of pathways that could explain the functions of IC50 of cisplatin, CCL18, and BCL2A1. Results indicate that the KEGG_TOLL_LIKE_RECEPTOR_SIGNALING_PATHWAY was associated with the sensitivity of COAD patients to cisplatin chemotherapy and immunotherapy. CCL18 and BCL2A1 might play a predictive role through the KEGG_TOLL_LIKE_RECEPTOR_SIGNALING_PATHWAY. Subgroup analysis confirmed that CC patients of immune C2, immune C6, HM-indel, HM-SNV, C18, C20, and High_CCL18_High_BCL2A1 were equally sensitive to cisplatin chemotherapy and immunotherapy.

## Materials and Methods

### Data Source

Gene expression data of CC samples were collected from public data sets at the TCGA (https://portal.gdc.cancer.gov/) and Gene Expression Omnibus (GEO) database (https://www.ncbi.nlm.nih.gov/geo/). For TCGA data, fragment per kilobase of transcript per million mapped reads (FPKM) was converted to TPM (transcript per kilobase of exon model per million mapped reads) and used in our study. Log2 transformation was used for normalization of GEO chip data (GSE39582 and GSE17538). After removing the missing values, our study included 419 TCGA-COAD patients, 566 GSE39582 CC patients, and 238 GSE17538 CC patients.

### Chemosensitivity Assessment

Based on transcriptome data of TCGA, GSE39582, and GSE17538 cohorts, the “pRRophetic” package was used to evaluate cisplatin chemotherapy sensitivity (Geeleher et al., 2014) and presented it in the form of the half maximal inhibitory concentration (IC50). According to the respective medians of cisplatin IC50, CC patients of each cohort were divided into low- and high-IC50 groups, respectively.

### The TME Score and Tumor Purity

The TME score enabled us to reflect the behavior and response of the cancer cells to a treatment process (Wang et al., 2018). A previous study proves that low tumor purity (the proportion of tumor cells in the TME) was associated with heavy mutation burden and intense immune phenotype in CC tissues (Mao et al., 2018). Estimation of STromal and Immune cells in MAlignant Tumor tissues using Expression data (ESTIMATE) is a method for estimating infiltrating nontumor cells (immune and stromal cells) in the TME based on gene expression profiles and two gene signatures (the immune and stromal signatures) (Wang H et al., 2019). Next, we inferred the purity of each patient’s tumor (Yoshihara et al., 2013).

### Quantification of TIICs

The TIIC abundance was a robust biomarker for immunotherapeutic response and immunophenotype determination (Zeng et al., 2020). The TIMER2.0 is a systematic platform for evaluations of the clinical influence of various TIICs in diverse cancer (Li T et al., 2020). Based on the TIMER2.0, we quantified the abundance of three types of innate TIICs and three types of adaptive TIICs in the CC microenvironment.

### Differentially Expressed Genes (DEGs)

For TCGA, DEGs between high- and low-IC50 samples were identifies using the “DEseq2” package (Kang et al., 2020). As a method of differential analysis of transcriptome count data, due to the shrinkage estimators for fold change (FC) and dispersion, DESeq2 improves the interpretability and stability of estimation (Love et al., 2014). For GSE39582 and GSE17538, DEGs between high- and low-IC50 tissues were identified using the “Limma” package. The adjusted *p*-value < .05 and |FC| > 2 were used as the cutoff criteria to filter DEGs (Wu et al., 2019).

### Receiver Operating Characteristic (ROC)

With the help of the “pROC” package (Robin et al., 2011), we built the ROC curve (high vs. low IC50, high vs. low PD-L1, and high vs. low pathway score).

### TMB

TMB is defined as the total number of gene encoding errors, base replacement, gene insertion or deletion errors per million bases (Schumacher et al., 2015). The 38 Mb is routinely taken based on the length of the human exon, so the TMB estimate for each sample is equal to the total mutation frequency/38 (Lv et al., 2020).

### Establishment of the Cisplatin-Resistant Cell Line DLD1

The resistant cell line DLD1 was established *in vitro* by intermittent exposure to different concentrations of cisplatin (S1166, selleck) in stepwise increments of time. Starting with a concentration of 5 μmol/L, cisplatin was added to the cells when they grew to ∼80% confluence. After 24 h, the remaining cells were cultured in cisplatin-free DMEM medium. When the surviving cells were restored to exponential growth, the next concentration of 5 FU (increase of 1 μmol/L) was then added. The cisplatin-resistant cell line DLD1 was established 9 months after the treatment was initiated, and the resistant phenotype was established.

### Measurement of Cell Viability

The CCK-8 assay was used to detect cell viability according to the manufacturer’s instructions. In short, DLD1 wild-type and cisplatin-resistance cells were cultured until ∼80% confluence, completely digested and added to each well (5000 cells/well) of a 96-well plate (Corning, United States). After 48 h of incubation, CCK-8 solution was added to each well of the 96-well plate. Finally, we used the microplate reader to read the OD value.

### RNA Isolation and Quantitative Reverse Transcription PCR (qRT-PCR)

The total RNA (DLD1 cell line and six pairs of CC tissues) was isolated according to the protocol of TRIZOL reagent (Life Technologies). The mRNA expressions of CCL18, BCL2A1, PD-L1, and β-actin were measured by the real-time PCR system (Applied Biosystems, Carlsbad, United States). The data were obtained by normalizing CCL18, BCL2A1, and PD-L1 gene Ct (cycle threshold) values with corresponding β-actin Ct and then analyzed with the 2-ΔΔCt Ct method (Xu et al., 2015). The primer sequences are as follows: CCL18 forward primer (5′-CTC​TGC​TGC​CTC​GTC​TAT​ACC-3′), CCL18 reverse primer (5′-CTT​GGT​TAG​GAG​GAT​GAC​ACC​T-3′), BCL2A1 forward primer (5′-TAC​AGG​CTG​GCT​CAG​GAC​TAT-3′), BCL2A1 reverse primer (5′-CGC​AAC​ATT​TTG​TAG​CAC​TCT​G-3′), PD-L1 forward primer (5′-TGG​CAT​TTG​CTG​AAC​GCA​TTT-3′), PD-L1 reverse primer (5′-TGC​AGC​CAG​GTC​TAA​TTG​TTT​T-3′), β-actin forward primer (5′-CAT​GTA​CGT​TGC​TAT​CCA​GGC-3′) and β-actin reverse primer (5′-CTC​CTT​AAT​GTC​ACG​CAC​GAT-3′).

### Correlation Diagram

The correlations between DEGs, PD-L1, and TME score were studied by Spearman’s correlation analysis and visualized using the “corrplot” and “PerformanceAnalytics” packages in R software.

### The Single-Sample GSEA (ssGSEA)

The 30 immune-related pathways (Shang et al., 2020) were retrieved on the GSEA website (https://www.gsea-msigdb.org/gsea/msigdb/genesets.jsp?collection=CC).

Using immune (Supplementary Table S1), autophagy (Supplementary Table S2), and ferroptosis (Supplementary Table S3) signatures, we quantified the immune, autophagy, and ferroptosis activities by the “GSVA” package and ssGSEA method (Hänzelmann et al., 2013).

### Heatmap

Heatmaps were constructed through the “pheatmap” package in R. Columns represent COAD tissues, and rows represent immune-related biomarkers. The levels of biomarkers were displayed in different colors, which transition from blue to red with increasing expression.

### Mutation Waterfall Charts

Somatic mutation data in the “masked somatic mutation” type was processed by VarScan2 (Koboldt et al., 2012). The “maftools” package was used to process and visualize the somatic mutation data of TCGA (Mayakonda et al., 2018).

### Autophagy-Related Genes (ARGs) and Ferroptosis-Related Genes

On the Human Autophagy Database (HADb, http://www.autophagy.lu/), we collected 232 ARGs (Supplementary Table S2) (Galluzzi et al., 2017). The ferroptosis-related genes were downloaded from FerrDb (Supplementary Table S3) (http://www.zhounan.org/ferrdb/) (Zhou and Bao, 2020).

### The UCSC XENA Database

On the UCSC XENA database (https://pancanatlas.xenahubs.net), an online exploration tool for public and private, multi-omic and clinical/phenotype data, we obtained TCGA-COAD patients’ immune subtype classification, molecular subtype classification, tumor stemness score based on RNAseq, icluster classification (Goldman et al., 2020), and visualized using R software.

### Functional Enrichment Analysis

To research the biofunctions of CCL18 and BCL2A1, the R “clusterProfiler” package (Yu et al., 2012) was used to perform functional annotations among 80 protein-coding genes with a correlation greater than 0.6 with CCL18 and BCL2A1, which included three categories of GO (biological processes (BP), molecular functions (MF), and cellular components (CC)) and KEGG enrichment analysis. Using the “treemap” package (Liu L et al., 2021), we visualized the results of functional enrichment analysis.

### The Encyclopedia of RNA Interactomes (ENCORI)

Using ENCORI (http://starbase.sysu.edu.cn/index.php), which is an open-source platform for studying miRNA–mRNA interactions, the miRNA differential expression and miRNA-target co-expression of CCL18, BCL2A1, and PD-L1 were examined.

### GSEA

The KEGG gene set (186 pathways) was downloaded from the MSigDB database (https://www.gsea-msigdb.org/gsea/msigdb/index.jsp) (Subramanian et al., 2005). Based on software GSEA_4.0.1, we performed the GSEA. Enrichment FDR values were based on 1000 permutations. Nominal *p*-value < .05 and FDR <0.25 were considered to be statistical significance (Cheng et al., 2021).

### Statistical Analysis

All statistical analyses in our study were performed using R software (version 4.0.3). Due to COAD transcriptome data, immune cell abundance, and ssGSEA scores do not follow a normal distribution, differences between two groups were tested by the Wilcoxon test. *p* < .05 was considered statistically significant: *p < .05, **p < .01, ***p < .001, ****p < .0001.

## Results

### Estimation of TME Score and TIIC Abundance

**FIGURE 1:**
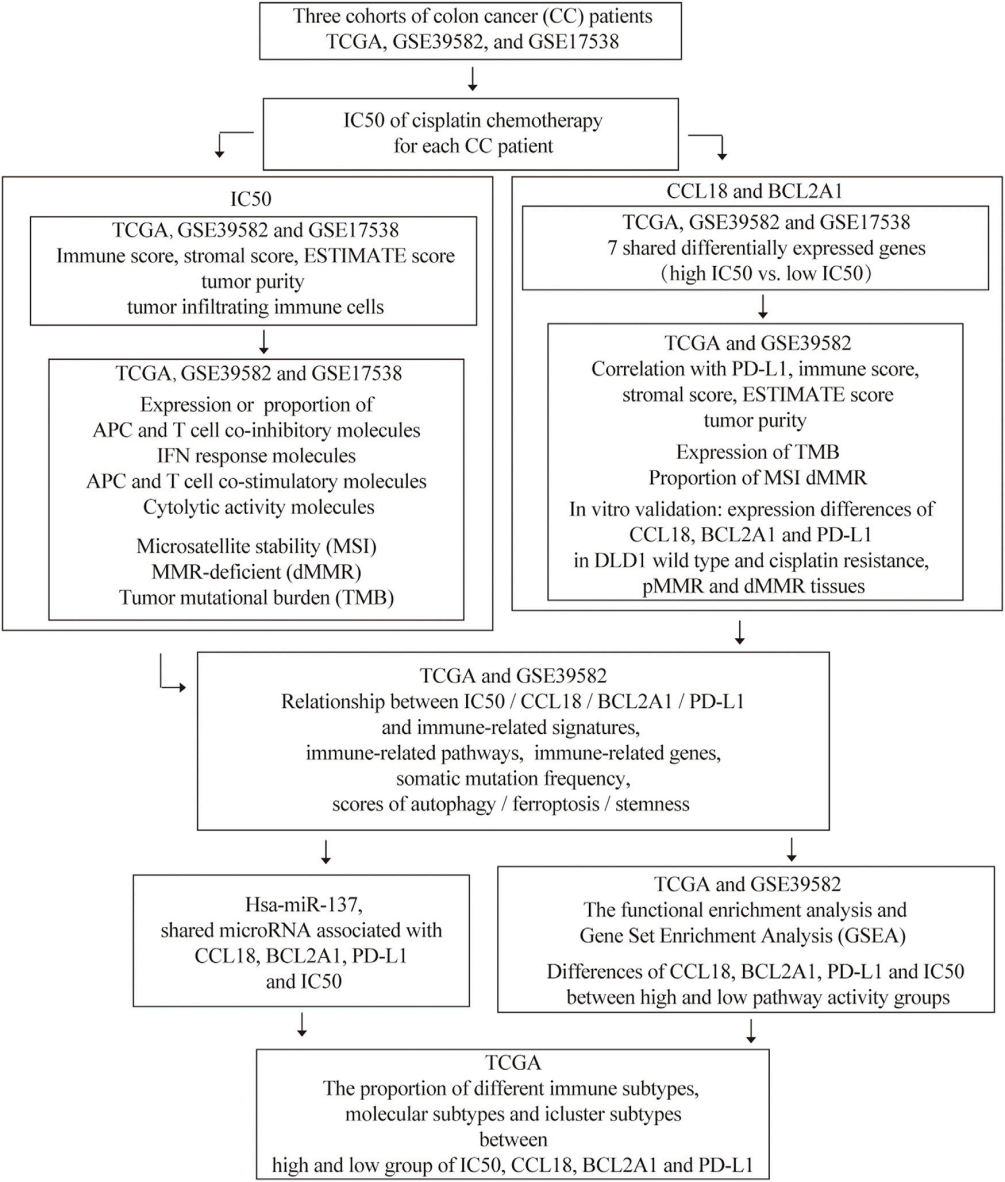
The flowchart of our research.

**FIGURE 2:**
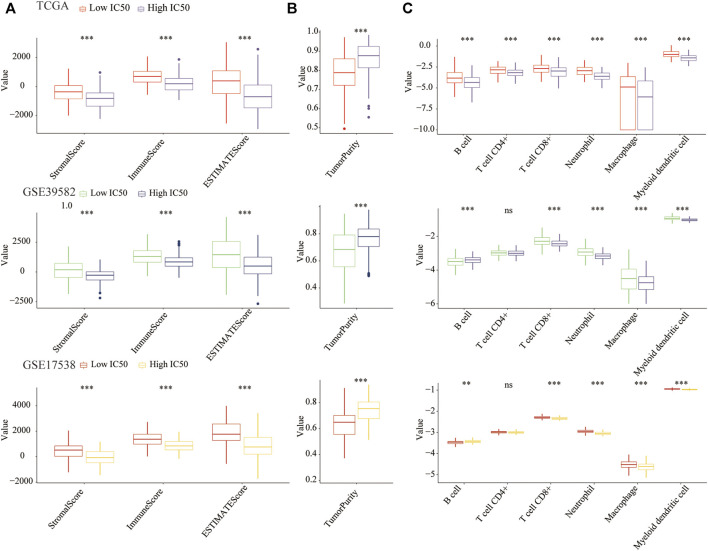
The COAD tissues of the low-IC50 group were more like hot tumors. **(A)** The stromal, immune, and ESTIMATE scores of the low-IC50 group were significantly higher than those of the low-IC50 group. **(B)** The tumor purity of CC tissue was significantly lower in the low-IC50 group. **(C)** Comparison of abundance of six kinds of tumor infiltrating immune cells between low- and high-IC50 groups. **p < .01, ***p < .001.

The flow chart of this research is shown in Figure 1. Previous studies show that the TME score and tumor purity (the proportion of tumor cells in the TME) were novel features to measure the efficacy of immunotherapy (Gong et al., 2020). Specifically, a high TME score or low-purity tumors exhibited a strong immunophenotype (Zhang et al., 2017). It was reported that cisplatin therapy could increase antitumor immune response by reducing immunosuppressive cells of the TME. Therefore, we studied the relationship between cisplatin chemotherapy and the TME in CC. Compared with the high-IC50 group, CC tissues in the low-IC50 group had higher stromal, immune, and ESTIMATE scores (Figure 2A) and lower tumor purity (Figure 2B), which suggests that the low-IC50 group are more like “hot tumors.” Furthermore, we investigated differences of the abundance of three types of innate TIICs and three types of adaptive TIICs in the TME. Combining the above three cohorts, the samples in the low-IC50 group had significantly higher infiltration of CD8+T cells, neutrophils, macrophages, and myeloid dendritic cells (MDCs) (Figure 2C). Therefore, we conclude that the immunophenotype of the low-IC50 group was different from that of the high-IC50 group and CC tissues sensitive to cisplatin chemotherapy were more like hot tumors and might be more sensitive to immunotherapy.

### Differences in the Expression of Immune-Related Genes

**FIGURE 3:**
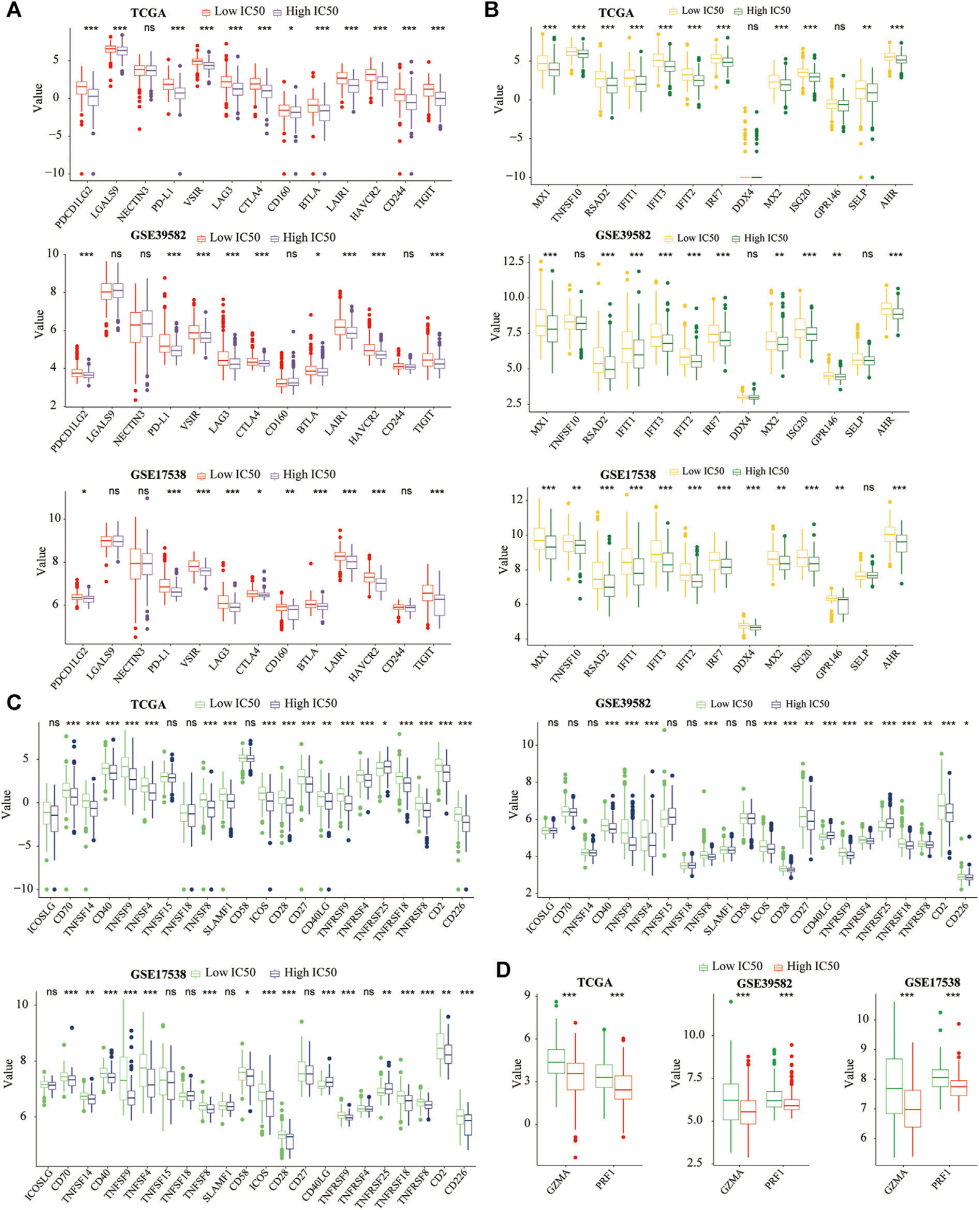
Differences of immune-related genes. The most **(A)** co-inhibitory molecules, **(B)** IFN response molecules, **(C)** co-stimulatory molecules, **(D)** cytolytic activity molecules were significantly higher in the low-IC50 group. *p < .05, **p < .01, ***p < .001.

To further clarify whether the low-IC50 group would benefit more from immunotherapy, we studied differences in the expression of co-inhibitory molecules of APC and T cells (Figure 3A), IFNG response molecules (Figure 3B), co-stimulatory molecules of APC and T cells (Figure 3C), and cytolytic activity molecules (Figure 3D) between low- and high-IC50 groups. The expression of most immune-related genes was significantly higher in the low-IC50 group, indicating that the CC in the low IC50 group had a stronger immunophenotype and would benefit more from immunotherapy.

### Other Predictive Biomarkers for Immunotherapy

**FIGURE 4:**
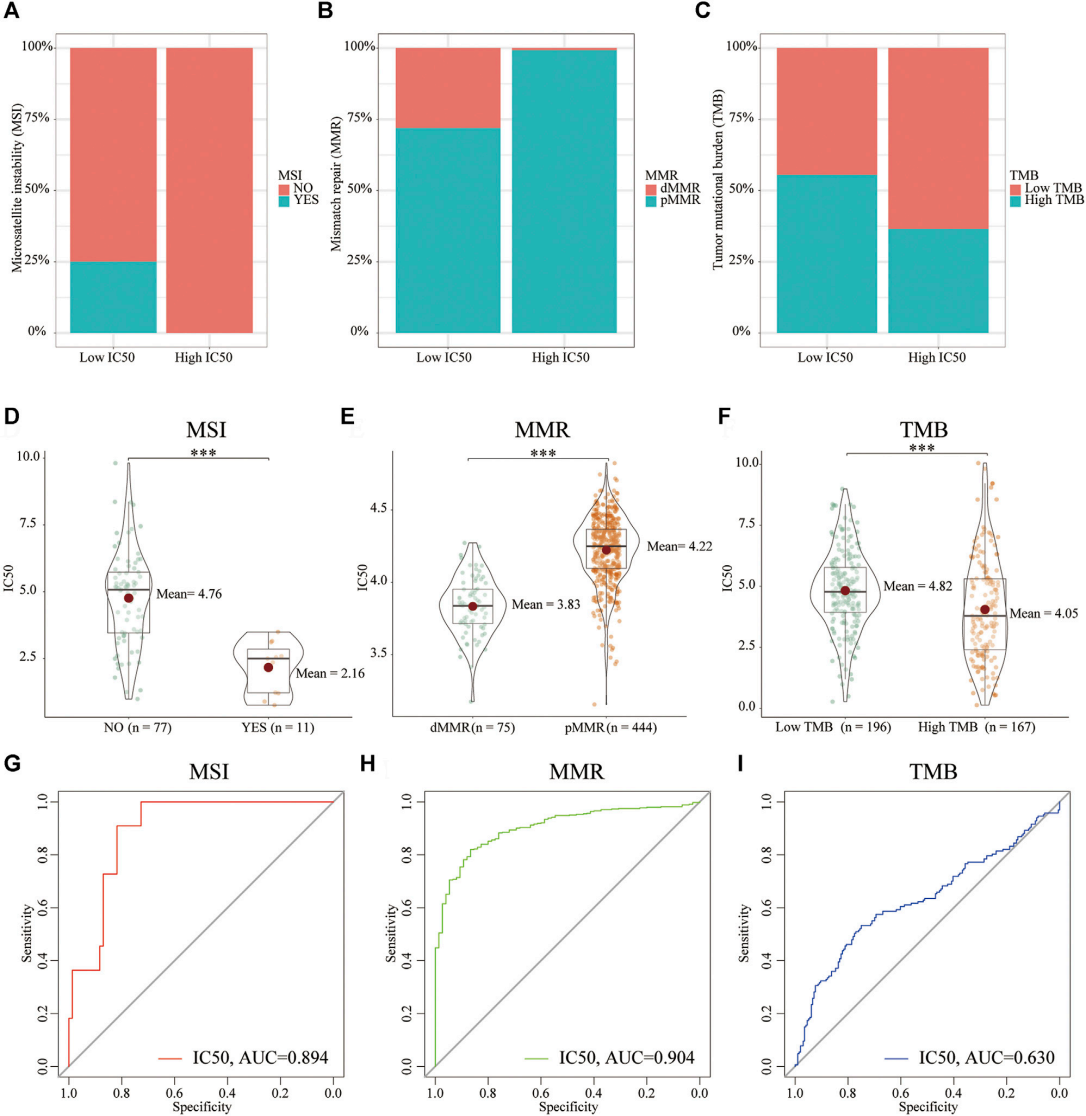
The relationship between hallmarks of genomic instability and IC50. Compared with the high-IC50 group, the low-IC50 group had a higher proportion of **(A)** MSI, **(B)** dMMR, and **(C)** more TMB. CC patients in the **(D)** MSI, **(E)** dMMR, **(F)** high TMB group had significantly lower IC50 for cisplatin chemotherapy. The IC50 had excellent diagnostic performance for **(G)** MSI and **(H)** dMMR,= and **(I)** general diagnostic performance for TMB. ***p < .001.

Next, we explored the relationship between the IC50 and other hallmarks of genomic instability (biomarkers for immunotherapy), including MSI, dMMR, and TMB (Halama et al., 2016; Duffy and Crown, 2019). Compared with the high-IC50 group, the low-IC50 group had a higher proportion of MSI (Figure 4A), dMMR (Figure 4B), and more TMB (Figure 4C). Similarly, patients of MSI, dMMR, and more TMB had lower IC50 (Figures 4D–F). The diagnostic power of cisplatin IC50 was evaluated using the area under the curve (AUC) of the ROC. The IC50 had excellent diagnostic performance for MSI (Figure 4G) and MMR (Figure 4H), and general diagnostic performance for TMB (Figure 4I). From this, we conclude that CC patients who were sensitive to cisplatin chemotherapy were more likely to be sensitive to immunotherapy.

### Cisplatin Resistance–Related Genes

**FIGURE 5:**
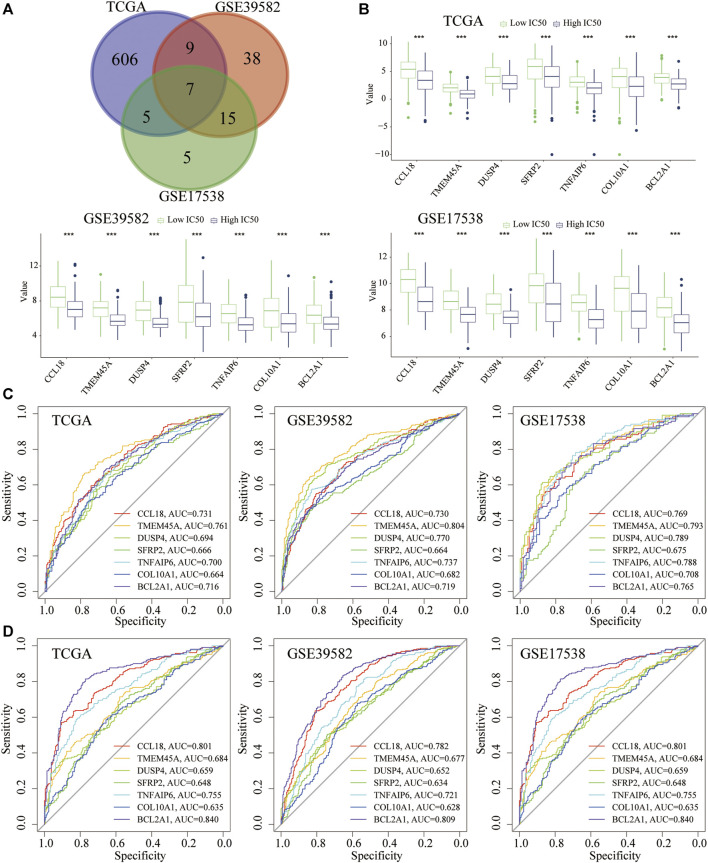
DEGs related to cisplatin resistance and their diagnostic performance. **(A)** The seven shared DEGs (High vs. low IC50). **(B)** The seven shared genes were significantly overexpressed in the low-IC50 group. **(C)** Diagnostic performance (high or low IC50) of seven genes. **(D)** Diagnostic performance (high or low PD-L1) of seven genes. ***p < .001.

To explore the transcriptomic signatures associated with cisplatin resistance, we conducted differential expression analysis between the low- and high-IC50 groups in three cohorts, respectively. Utilizing the DEseq2 (TCGA) and limma (GSE39582 and GSE17538) algorithms, a total of seven shared DEGs were screened (Figure 5A), all of which were downregulated in the high-IC50 group (Figure 5B). Diagnostic power (high vs. low IC50) (high vs. low PD-L1) of these seven genes were also evaluated using the AUC of the ROC. Combining three data sets, for the quantification of IC50, CCL18, TMEM45A, TNFAIP6, and BCL2A1 had higher diagnostic efficiency (AUC > 0.7) (Figure 5C). For the quantification of PD-L1 expression (biomarker in cancer immunotherapy), CCL18, TNFAIP6, and BCL2A1 had higher diagnostic efficiency (AUC > 0.7) (Figure 5D). We concluded that the mechanisms for the low-IC50 group to benefit from immunotherapy might be closely related to the increased expression of CCL18, TNFAIP6, and BCL2A1.

### Correlation of Cisplatin Resistance–Related Genes With PD-L1 Expression and the TME Score

**FIGURE 6:**
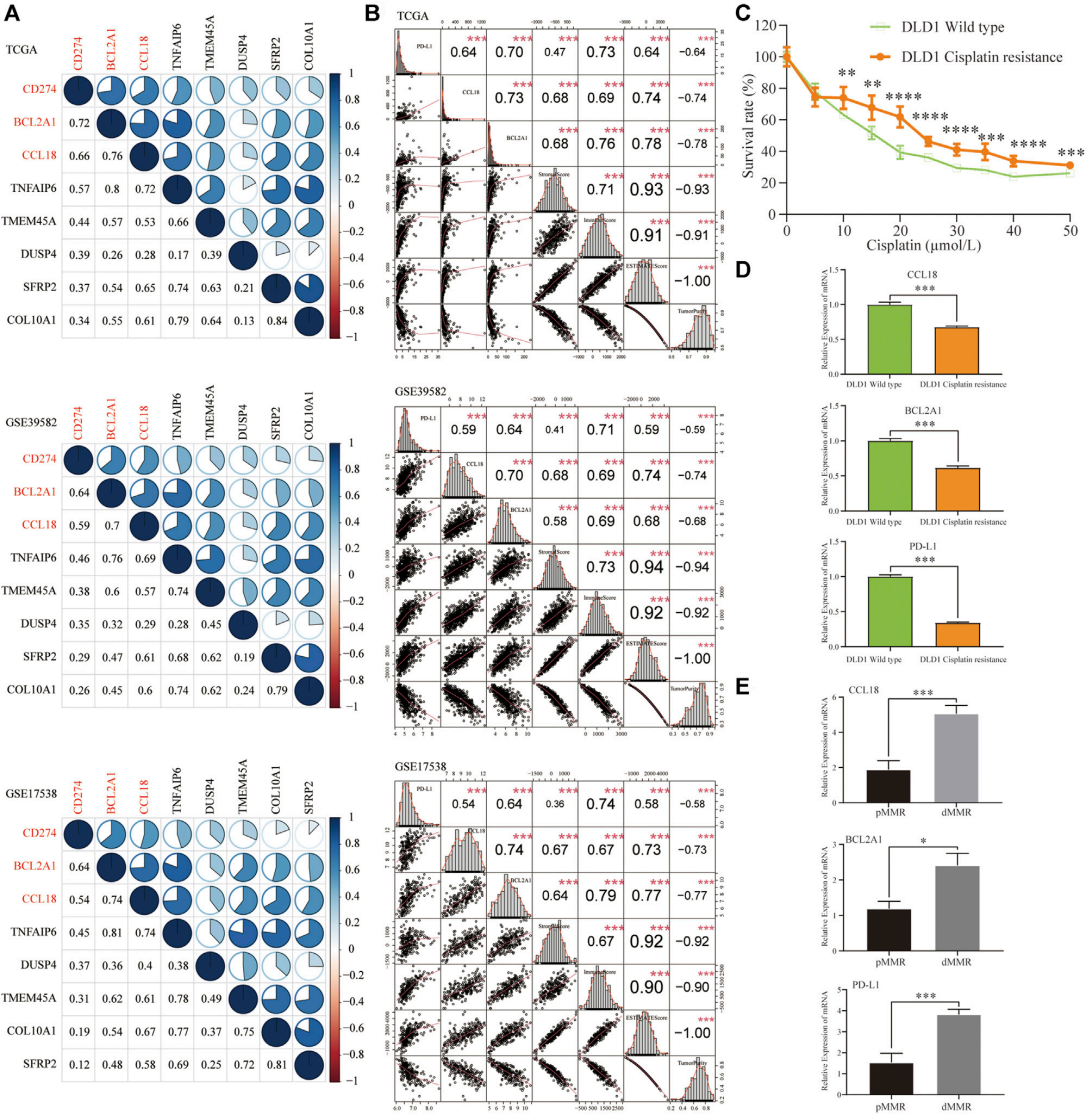
Correlation of seven resistance-related genes with PD-L1 expression, the TME scores, tumor purity, and *in vitro* validation. In three COAD cohorts, **(A)** CCL18 and BCL2A1 had a higher correlation with PD-L1 (Spearman’s correlation coefficient >0.5) **(B)** Similar to PD-L1, CCL18 and BCL2A1 were significantly positively correlated with stromal, immune, and ESTIMATE scores and significantly negatively correlated with tumor purity. In cisplatin-resistant DLD1 cell line **(C)**, similar to PD-L1, CCL18 and BCL2A1 were significantly downregulated **(D)**. **(E)** Compared with pMMR tissues, the mRNA expression of CCL18, BCL2A1 and PD-L1 in dMMR was significantly increased. **p < .01, ***p < .001, ****p < .0001.

In the above three cohorts, we further performed Spearman correlation analysis among PD-L1 and seven cisplatin resistance–related genes. Taking the correlation coefficient greater than 0.5 as the threshold, CCL18 and BCL2A1 were significantly associated with PD-L1 (Figure 6A). Besides this, similar to PD-L1, CCL18 and BCL2A1 were also significantly positively correlated with TME score (stromal, immune, and ESTIMATE scores), whereas negatively associated with tumor purity (Figure 6B). It could be seen that the expression of cisplatin resistance–related genes, CCL18 and BCL2A1, were significantly related to the immunophenotype of the TME.

Compared with DLD1 wild-type, the survival rate of drug-resistant DLD1 increased significantly when the concentration of cisplatin was more than 10 µmol/L (Figure 6C). For *in vitro* verification, compared with DLD1 wild-type, the expression of CCL18, BCL2A1, and PD-L1 was significantly decreased in cisplatin-resistant DLD1 (Figure 6D). Next, we evaluated the CCL18, BCL2A1, and PD-L1 mRNA expression in six pairs CC tissues (pMMR vs. dMMR) of our hospital. The patient’s consent was obtained in advance, and informed consent was signed. Compared with pMMR tissues, the mRNA expression of CCL18, BCL2A1, and PD-L1 in dMMR was significantly increased (Figure 6E). The *in vitro* wet experiments were consistent with the abovementioned bioinformatics analysis conclusion, that is, CC tissues that were sensitive to cisplatin chemotherapy might be more sensitive to immunotherapy.

### The Relationship Between CCL18, BCL2A1, and Hallmarks of Genomic Instability

**FIGURE 7:**
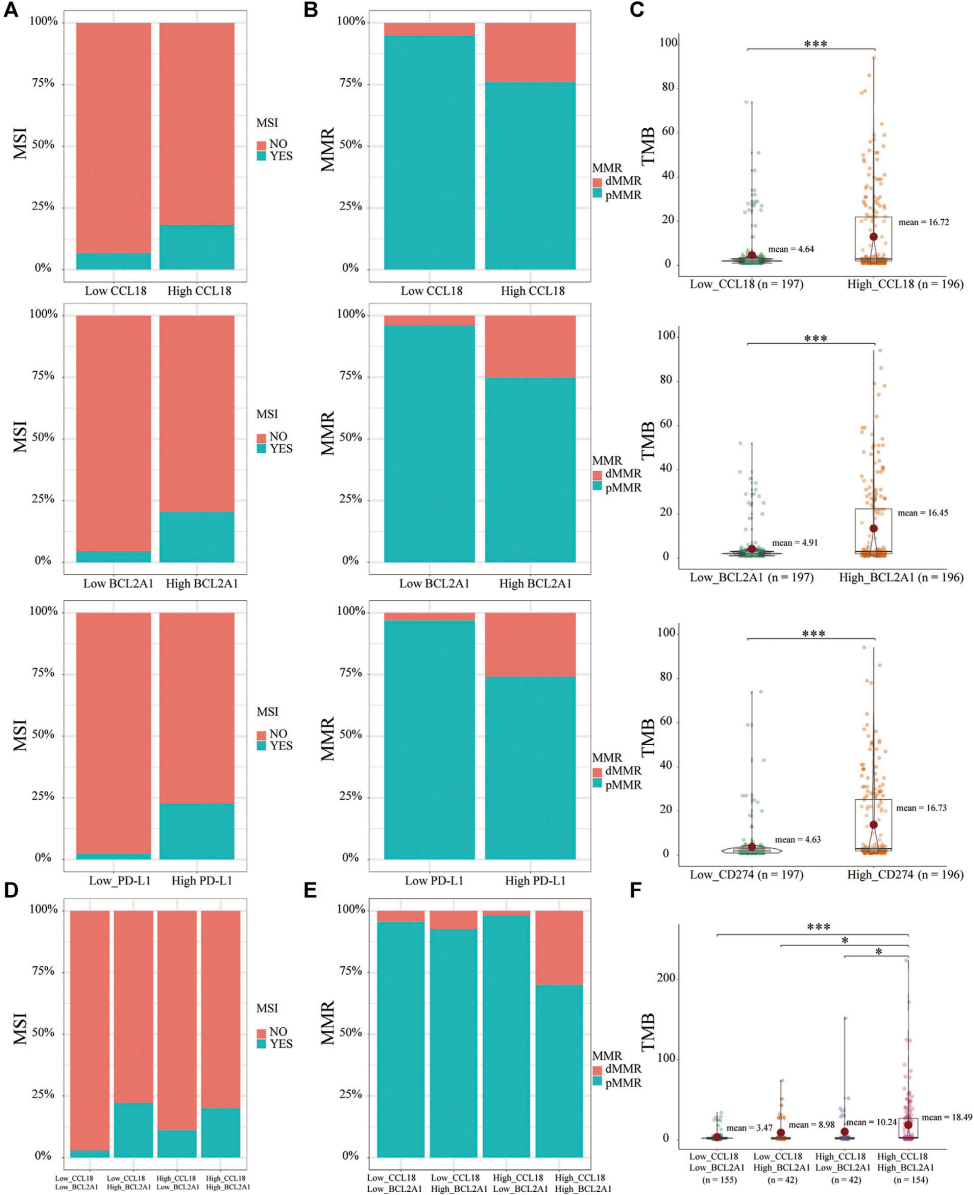
The relationship between MSI, dMMR, TMB, and CCL18, BCL2A1. Similar to PD-L1, the high CCL18 or BCL2A1 group had a greater proportion of **(A)** MSI, **(B)** dMMR, and **(C)** more TMB. **(D)** Low_CCL18_High_BCL2A1 and High_CCL18_High_BCL2A1 groups had a greater proportion of MSI. Compared with the other three groups, the High_CCL18_High_BCL2A1 group had the highest proportion of **(E)** dMMR and the most **(F)** TMB. *p < .05, ***p < .001.

To further determine whether CCL18 and BCL2A1 could be used as predictive biomarkers for cancer immunotherapy, such as cisplatin IC50, we investigated their relationship with hallmarks of genomic instability (biomarkers for immunotherapy). In contrast with the low group of CCL18, BCL2A1, and PD-L1, the corresponding high group had a higher proportion of MSI (Figure 7A), dMMR (Figure 7B), and more TMB (Figure 7C).

Based on respective medians of CCL18 and BCL2A1, CC patients were divided into four groups, including Low_CCL18_Low_BCL2A1, Low_CCL18_High_BCL2A1, High_CCL18_Low_BCL2A1 and High_CCL18_High_BCL2A1. Low_CCL18_High_BCL2A1 and High_CCL18_High_BCL2A1 patients had a higher proportion of MSI (Figure 7D). BCL2A1 was better than CCL18 in terms of MSI prediction. For MMR, High_CCL18_High_BCL2A1 patients had the highest proportion of dMMR (Figure 7E). For TMB, High_CCL18_High_BCL2A1 patients had the highest TMB (Figure 7F). Therefore, compared with the other three groups, High_CCL18_High_BCL2A1 patients would benefit more from chemotherapy or immunotherapy. Besides this, For MSI prediction, BCL2A1 had a better prediction of the efficacy of immunotherapy. Based on the expression of CCL18 and BCL2A1, we could partly predict the sensitivity of immunotherapy in CC patients.

### Scores of Immune Signatures and Immune Pathways and Expression of Immunomodulators

**FIGURE 8:**
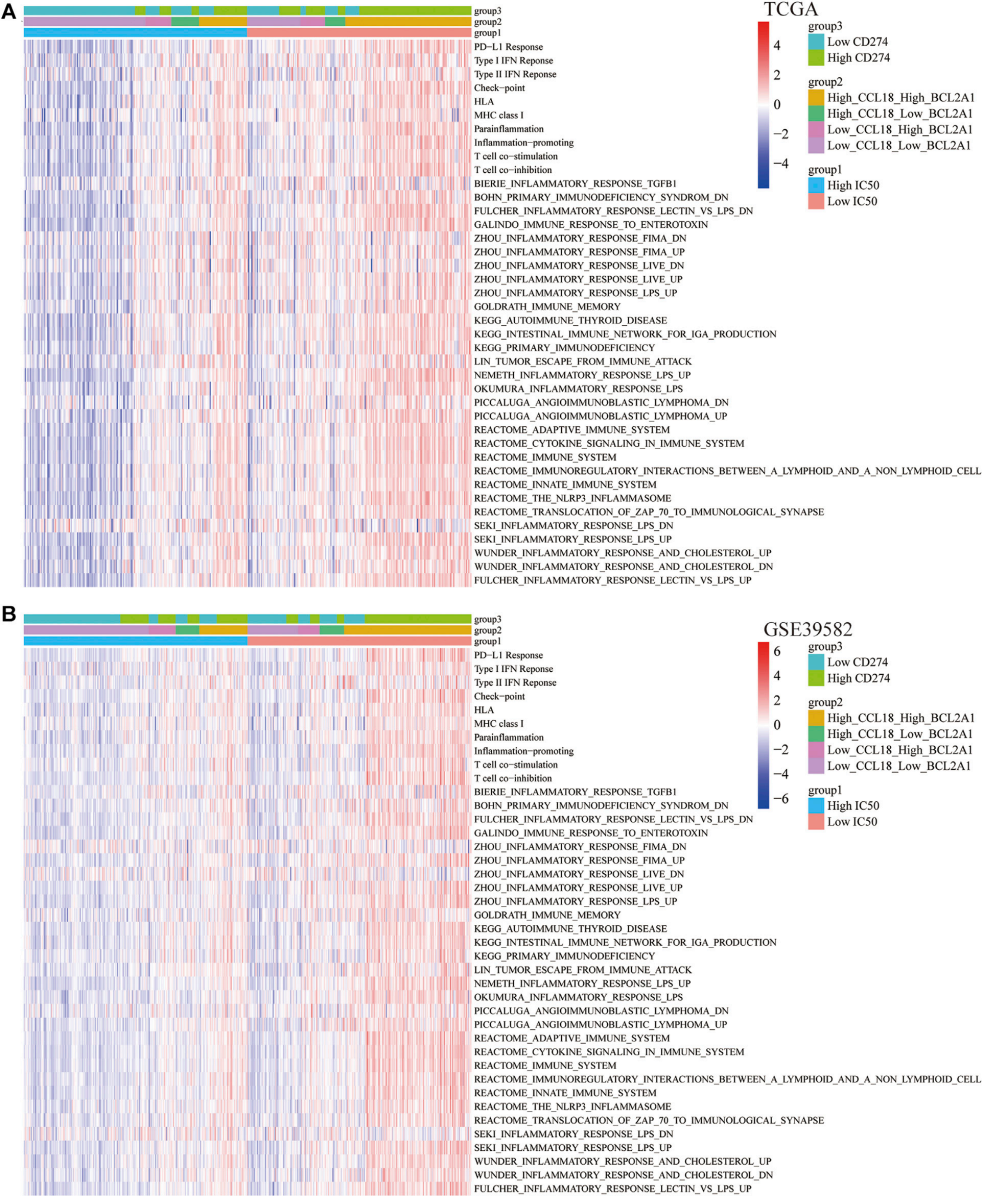
Correlation of immune signatures and pathways. In **(A)** TCGA and **(B)** GSE39582, the score of most immune signatures and pathways were significantly higher in the low-IC50 group and High_CCL18_High_BCL2A1 group.

**FIGURE 9:**
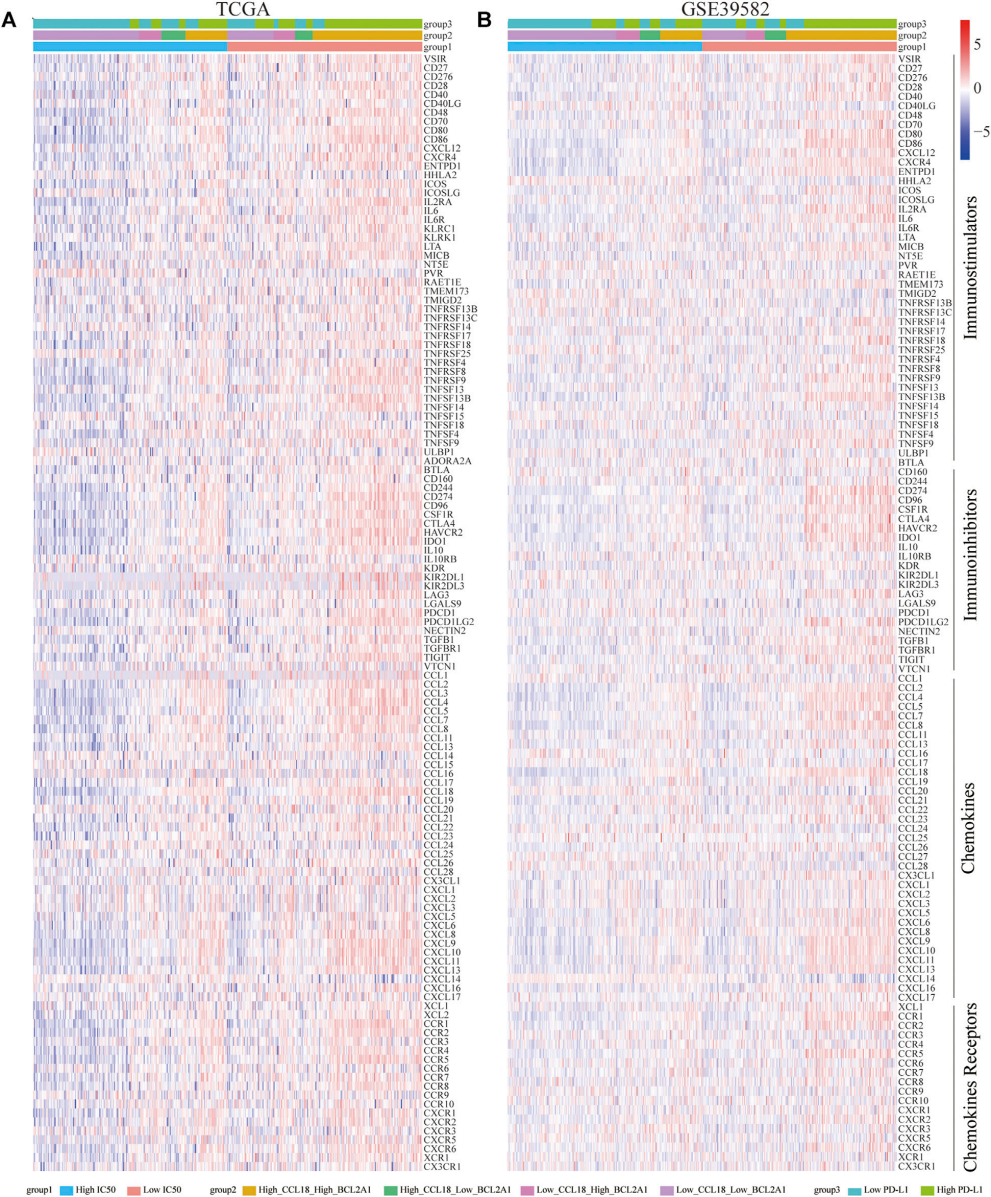
Correlation of immunomodulators (immunostimulators, immunoinhibitors, chemokines, and chemokine receptors). In **(A)** TCGA and **(B)** GSE39582, most immunomodulators were significantly upregulated in the low-IC50 group and High_CCL18_High_BCL2A1 group.

Based on the ssGSEA algorithm, we calculated the scores of 10 immune signatures and 30 immune pathways and further forecasted their relationship with cisplatin IC50, CCL18, BCL2A1, and PD-L1. Results indicate that, with the increasing expression of CCL18 and BCL2A1, most immune responses were activated as well as immune-related pathways (Figures 8A,B). Besides this, for immunomodulators, whether it was immunostimulators, immunoinhibitors, chemokines, or chemokine receptors, most of them were significantly positively correlated with the expression of CCL18 and BCL2A1 (Figures 9A,B). Cisplatin resistance–related genes CCL18 and BCL2A1 could partly predict the efficacy of immunotherapy in CC patients.

### The Landscapes of Gene Mutations

**FIGURE 10:**
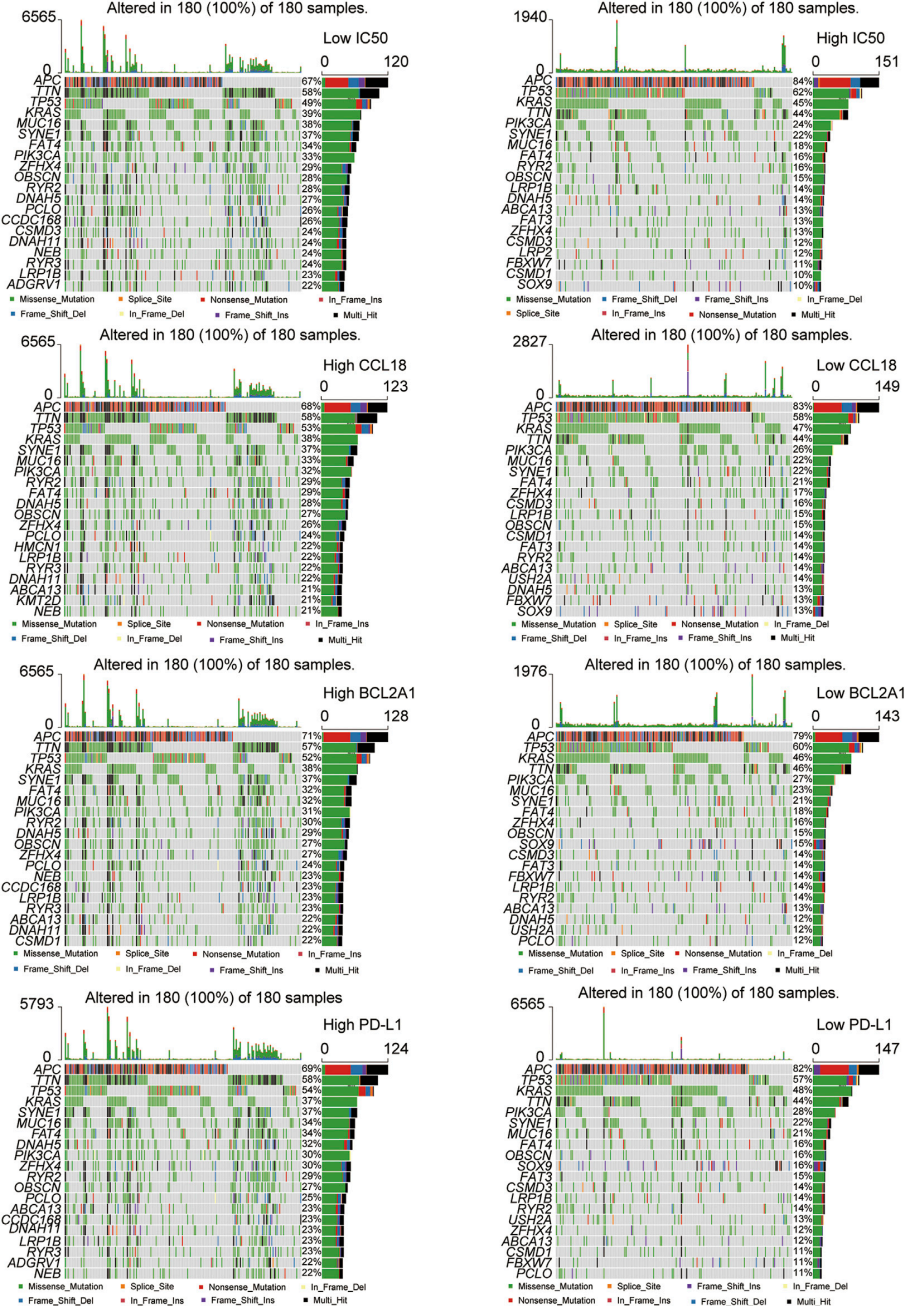
The landscapes of somatic mutations. The low-IC50 group and the high CCL18 or BCL2A1 or PD-L1 group had more somatic mutation frequencies.

Gene mutations caused cancer patients to be sensitive or resistant to immune drugs, affecting clinical drug selection and treatment effects (Peng et al., 2016). Therefore, we investigated the landscapes of gene mutations in the low and high groups of TCGA-COAD patients. Similar to the PD-L1 high group, for the low-IC50 group and high-CCL18 or BCL2A1 group, among the 20 most frequently mutated genes, there were more TTN, MUC16, SYNE1, FAT4, PIK3CA, ZFHX4, OBSCN, RYR3, DNAH5, PCLO, DNAH11, NEB, and LRP1B mutation and less APC, TP53, and KRAS mutation (Figure 10). It could be seen that gene mutation frequencies were closely related to immunotherapy response in CC patients.

### Difference of Autophagy, Ferroptosis, and Stemness Scores

**FIGURE 11:**
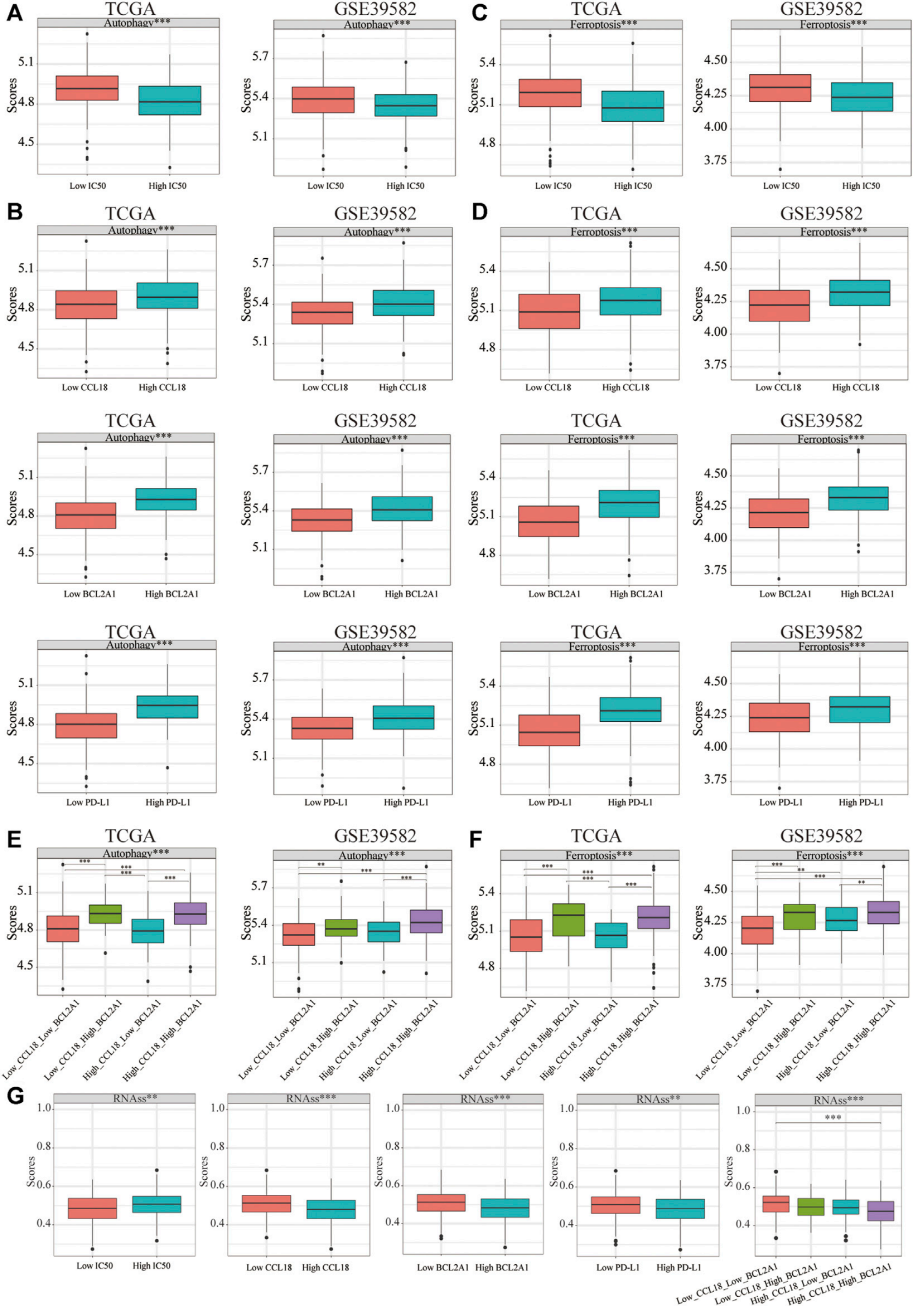
The difference of autophagy, ferroptosis, and stemness scores. In TCGA and GSE39582, the autophagy score of **(A)** the low-IC50 group and **(B)** high group of CCL18 or BCL2A1 or PD-L1 were significantly higher. Similarly, for ferroptosis, the score of **(C)** the low-IC50 group and **(D)** high group of CCL18 or BCL2A1 or PD-L1 were significantly higher. **(E**,**F)** The autophagy or ferroptosis score of High_CCL18_High_BCL2A1 patients were significantly higher than that of Low_CCL18_Low_BCL2A1 and High_CCL18_Low_BCL2A1 patients. The autophagy or ferroptosis score of Low_CCL18_High_BCL2A1 patients were significantly higher than that of Low_CCL18_Low_BCL2A1 patients. **(G)** The stemness score of the low IC50 group and high group of CCL18 or BCL2A1 or PD-L1 was significantly upregulated. The stemness score of High_CCL18_High_BCL2A1 patients was significantly upregulated than that of Low_CCL18_Low_BCL2A1 patients. **p < .01, ***p < .001.

Previous studies confirm that autophagy mediated the sensitivity of tumor cells to immunotherapy after chemotherapy (Ramakrishnan et al., 2012). In our study, in the TCGA and GSE39582 data sets, compared with the low-IC50 group, the autophagy score of the high-IC50 group was significantly decreased (Figure 11A), which was consistent with previous study that autophagy activators could inhibit the cisplatin resistance of tumor cells (Sirichanchuen et al., 2012). Compared with the low group of CCL18 or BCL2A1 or PD-L1, the autophagy score of corresponding high group was significantly increased (Figure 11B), which was consistent with the conclusions of previous studies that enhanced autophagy would increase the sensitivity of immunotherapy (Jiang et al., 2019; Xia et al., 2021).

Ferroptosis, the bright new star in immunotherapy, could enhance the efficacy of immunotherapy (Wang W et al., 2019). Besides this, ferroptosis inducers could enhance chemotherapeutic drug sensitivity (Kim et al., 2018). Similar to the difference in autophagy score, the low-IC50 group and the high-CCL18 or BCL2A1 or PD-L1 group had higher ferroptosis scores (Figures 11C,D). Among the four groups, the autophagy or ferroptosis score of High_CCL18_High_BCL2A1 patients were significantly higher than that of Low_CCL18_Low_BCL2A1 and High_CCL18_Low_BCL2A1 patients. The autophagy or ferroptosis score of Low_CCL18_High_BCL2A1 patients were significantly higher than that of Low_CCL18_Low_BCL2A1 patients (Figures 11E,F). The difference between autophagy and ferroptosis further verified that CCL18 and BCL2A1 were closely related to the sensitivity of immunotherapy in CC patients.

**FIGURE 12:**
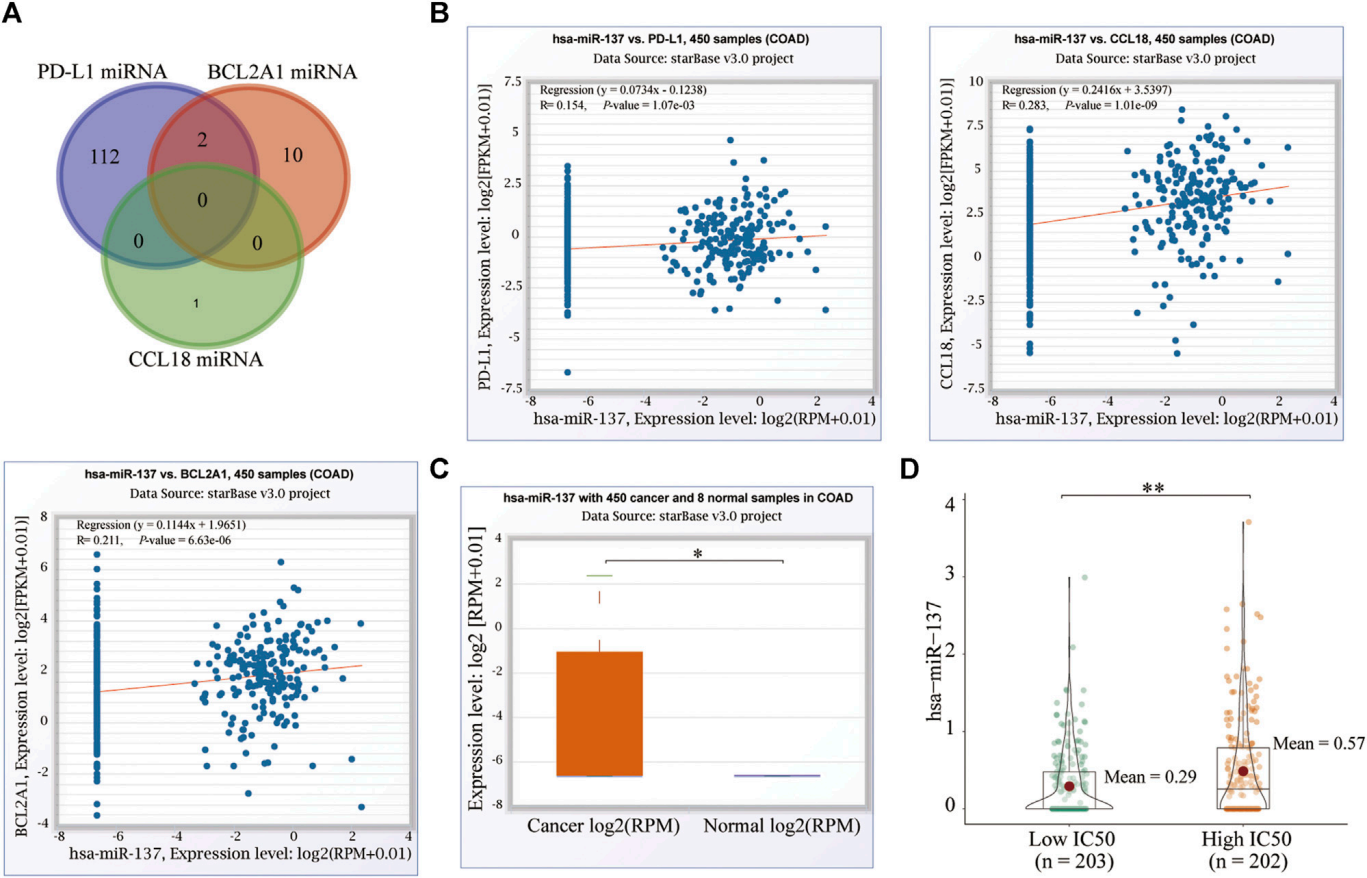
MicroRNAs correlation and **(A)** shared miRNAs of PD-L1, CCL18, and BCL2A1. **(B)** One of the common miRNAs of PD-L1 and BCL2A1, hsa-miR-137, was significantly related to PD-L1, BCL2A1, and CCL18. **(C)** Hsa-miR-137 was significantly upregulated in CC tissues and the highIC50 group. *p < 0.05, **p < .01.

Previous study confirms that the stemness of immune T cells promotes antitumor effects (Li W et al., 2020). Therefore, we analyzed the stemness score difference between low and high IC50 or CCL18 or BCL2A1 or PD-L1 groups. (Figure 11G) The high-IC50 group and low-CCL18 or BCL2A1 or PD-L1 group had a higher stemness score. The stemness score of High_CCL18_High_BCL2A1 patients was significantly lower than that of Low_CCL18_Low_BCL2A1 patients. Therefore, we speculated that the outcome of chemotherapy and immunotherapy for CC patients with high stemness score might not be good (Figure 12).

### MicroRNA Correlation

The mechanism of drug resistance is very extensive in cytology, including a variety of key genes and pathways. The genetic variation of these key factors can lead to the occurrence of drug resistance in tumor cells. Among them, microRNA is one of these key genes (Ma et al., 2010). On the ENCORI database, we excavated the respective miRNAs of CCL18, BCL2A1, and PD-L1 and finally screened the two miRNAs shared by BCL2A1 and PD-L1, hsa-miR-514a-5p and hsa-miR-137 (Figure 12A). Hsa-miR-137 was significantly positively associated with CCL18, BCL2A1, and PD-L1 (Figure 12B), and upregulated in CC (Figure 12C) and the high-IC50 group (Figure 12D). Previous studies confirm that hsa-miR-137 was closely related to the occurrence and development (Smith et al., 2015; Huang et al., 2016), chemotherapy sensitivity (Guo et al., 2017) of cancer, and could also regulate the autophagy (Wang Z et al., 2019), ferroptosis (Luo et al., 2018), and stemness (Sakaguchi et al., 2016) of cancer cells. To a certain extent, this confirms the conclusions of our analysis. However, the roles of hsa-miR-137 in cancer immunotherapy were never mentioned, which needed to be explored in depth. For the other microRNA, hsa-miR-514a-5p was only positively significantly associated with BCL2A1. Therefore, we did not study it further.

### The Functional Enrichment Analysis (GO and KEGG) and GSEA

**FIGURE 13:**
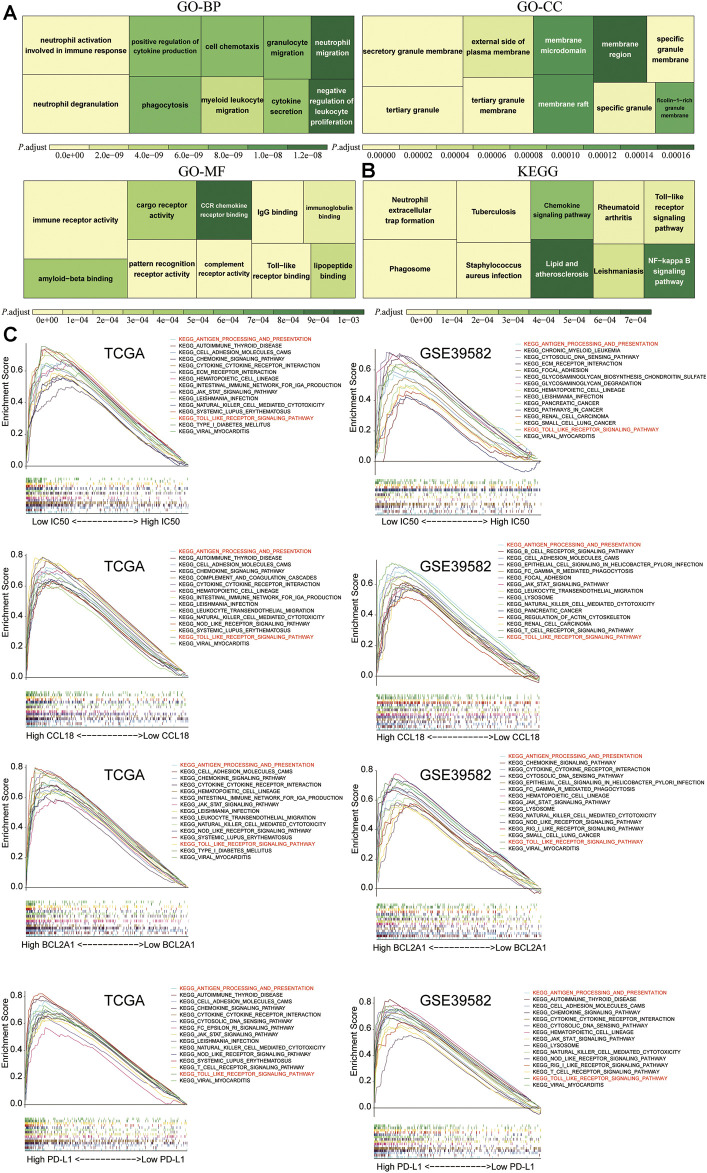
The functional enrichment analysis and GSEA. The **(A)** GO and **(B)** KEGG analysis of 80 genes significantly related to CCL18 and BCL2A1 (Spearman’s correlation coefficient >0.6) in TCGA and GSE39582. **(C)** In TCGA and GSE39582, between the high and low groups of IC50, CCL18, BCL2A1, and PD-L1, among 186 KEGG pathways, there were two shared significant enrichment pathways, which are marked in red.

To explore the underlying mechanisms of CCL18 and BCL2A1, among 19,584 protein-coding genes in the TCGA and 20,183 protein-coding genes in the GSE39582, based on the Spearman correlation coefficient greater than 0.6, we unearthed 80 genes related to both CCL18 and BCL2A1 and performed GO (Figure 13A) and KEGG (Figure 13B) analysis. For BP, MF, and KEGG, CCL18 and BCL2A1 were significantly related to immune and inflammatory response. For CC, CCL18 and BCL2A1 were significantly related to granule membrane. Besides this, results of GO-MF and KEGG were both significantly related to the function of Toll-like receptors. Toll-like receptors might be involved in the resistance process of chemotherapy and immunotherapy in CC patients.

To further study potential pathways for chemotherapy-sensitive tumor cells to benefit from immunotherapy and the molecular mechanisms of CCL18 and BCL2A1, we performed GSEA among 186 KEGG pathways, including the Toll-like receptor signaling pathway. Results indicate that the low-IC50 group and high-CCL18 or BCL2A1 or PD-L1 group were significantly associated with KEGG_ANTIGEN_PROCESSING_AND_PRESENTATION and KEGG_TOLL_LIKE_RECEPTOR_SIGNALING_PATHWAY (Figure 13C). Downregulating the activity of these two pathways might help increase the sensitivity of drug-resistant tumor cells to cisplatin chemotherapy and immunotherapy.

### Differences in the Scores of two Enrichment Pathways

**FIGURE 14:**
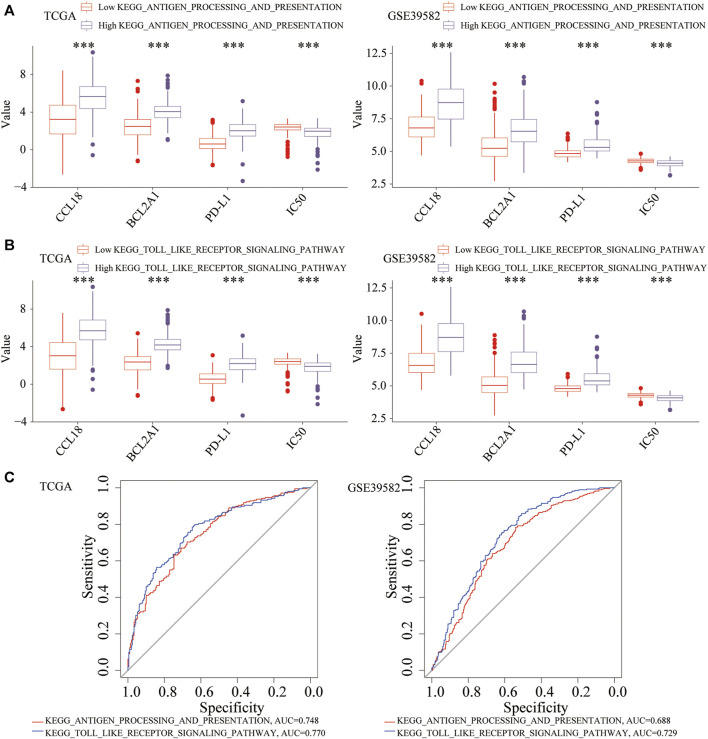
The relationship between two key pathways and the expression of CCL18, BCL2A1, PD-L1, and IC50. In the high **(A)** KEGG_ANTIGEN_PROCESSING_AND_PRESENTATION or **(B)** KEGG_TOLL_LIKE_RECEPTOR_SIGNALING_PATHWAY group, CCL18, BCL2A1 and PD-L1 were significantly upregulated, and the IC50 was significantly downregulated. **(C)** Diagnostic efficacy of the two pathways (high vs. low IC50). ***p < .001.

Using the ssGSEA method, we quantified the scores of the above two key pathways. Based on the respective medians of the pathway score, CC patients were divided into high and low groups. In the high group of two key pathways, the expression of CCL18, BCL2A1, and PD-L1 increased significantly, whereas the IC50 decreased significantly (Figures 14A,B). Diagnostic power (high vs. low IC50) of two pathways were also evaluated using the AUC of the ROC. The diagnostic power of KEGG_TOLL_LIKE_RECEPTOR_SIGNALING_PATHWAY was higher than KEGG_ANTIGEN_PROCESSING_AND_PRESENTATION (Figures 14C). This corresponded to the Toll-like receptor function of the previous GO-MF and KEGG results. The Toll-like receptor signaling pathway might play a pivotal role in the response to chemotherapy and immunotherapy and was a key target to increase treatment sensitivity.

### Subtype Analysis of TCGA-COAD Patients

**FIGURE 15:**
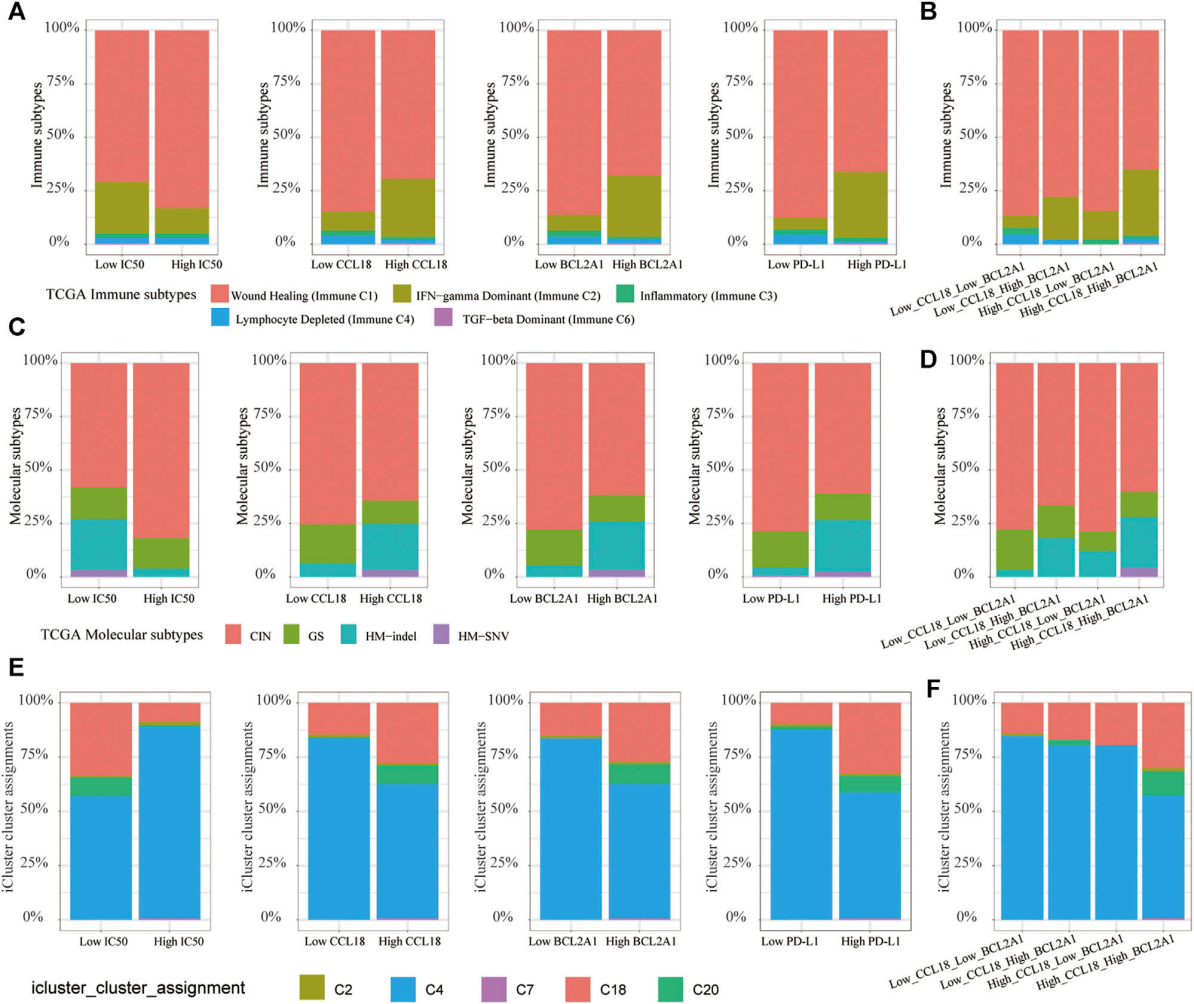
TCGA-COAD subtype analysis. **(A**,**B)** The proportion of immune C2 and C6 increased significantly in the low-IC50 group, high CCL18 or BCL2A1 or PD-L1 group, and High_CCL18_High_BCL2A1 group. **(C**–**F)** The low-IC50 group, high group of CCL18 or BCL2A1 or PD-L1 and Low_CCL18_Low_BCL2A1 group had a higher proportion of HM-indel, HM-SNV, C18 and C20.

To understand the pathogenesis of fatal malignant tumors, 33 types of tumors in the TCGA were classified into different subtypes according to the genomic characteristics (Liu et al., 2018; Thorsson et al., 2018; Swanson et al., 2019). We explored the proportion of different immune subtypes, molecular subtypes and icluster subtypes of a TCGA-COAD cohort. Among five types of immune subtypes of TCGA-COAD, the proportion of IFN-γ dominant (immune C2) and TGF-beta dominant (Immune C6) patients in the low-IC50 group, high CCL18 or BCL2A1 or PD-L1 group, and High_CCL18_High_BCL2A1 group increased significantly (Figures 15A,B), indicating that these patients were suitable for cisplatin chemotherapy and immunotherapy. Among four types of molecular subtypes of TCGA-COAD, patients of HM-indel and HM-SNV might benefit more from cisplatin chemotherapy and immunotherapy (Figures 15C,D). Using the iCluster and Cluster of Cluster Assignments (COCAs) method (Swanson et al., 2019), CC patients were mainly divided into five types, of which C18 and C20 patients were mainly attributable to low-IC50 group, high-CCL18 or BCL2A1 or PD-L1 group, and High_CCL18_High_BCL2A1 group (Figures 15E,F). It could be seen that C18 and C20 patients might benefit more from cisplatin chemotherapy and immunotherapy.

## Discussion

As one of the leading causes of cancer-related death, the treatment of COAD has always been highly concerned. The immune TME occupies an important position in the process of tumor occurrence, invasion, metastasis, and treatment tolerance, indicating that we can start from the perspective of the immune microenvironment to explore novel ant-tumor targets.

With the increasing popularity of immunotherapy, it has brought hope to cancer patients. However, not all patients can respond well to immunotherapy (Ganesh et al., 2019). Therefore, the development of immune-related biomarkers with high accurate value is essential to predict the efficacy of immunotherapy in COAD patients.

In this study, we found that CCL18 and BCL2A1 could predict the efficacy of chemotherapy and immunotherapy at the same time. At the subtype level, COAD patients of immune C2, immune C6, HM-indel, HM-SNV, C18 and C20 were suitable for chemotherapy and immunotherapy. At the genetic level, High_CCL18_High_BCL2A1 patients might benefit the most from chemotherapy and immunotherapy. Low_CCL18_High_BCL2A1 patients had a high proportion of MSI, which was close to that of High_CCL18_High_BCL2A1 patients. In addition, the proportion of immune C2 and HM-indel in Low_CCL18_High_BCL2A1 patients was higher than that of High_CCL18_Low_BCL2A1. From this, we concluded that BCL2A1 was better than CCL18 in predicting the sensitivity of COAD patients to chemotherapy and immunotherapy.

To study the potential mechanisms of CCL18 and BCL2A1, GSEA and functional enrichment analysis were conducted. Results show that the expression changes of CCL18 and BCL2A1 mainly affected the Toll-like receptor signaling pathway, which provides clues for further research.

For hsa-miR-137, a previous study confirmed its correlation with chemotherapy sensitivity. Hsa-miR-137 chemosensitized CC cells to the chemotherapeutic drug oxaliplatin by targeting YBX1 (Guo et al., 2017). Silencing OIP5-AS1 and upregulating hsa-miR-137 expression significantly intensified growth inhibition of drug-resistant CC cells and improved the sensitivity of CC cells to Oxaliplatin (Liang et al., 2020). Besides this, hsa-miR-137 was closely related to the occurrence of early colorectal cancer (Huang et al., 2016). Hsa-miR-137 acted as a tumor-suppressive miRNA in CCs and negatively regulated the progression of CC (Smith et al., 2015). Hsa-miR-137 was also closely related to ferroptosis (Luo et al., 2018), stemness (Sakaguchi et al., 2016), and autophagy (Wang Z et al., 2019) of cancer cells. However, the roles of hsa-miR-137 in immunotherapy had never been explored, and more molecular biology experiments were urgently needed.

CCL18 is a member of the secreted protein cytokine family involved in immunoregulatory and inflammatory processes (Babu et al., 2009). BCL2A1 is the underdog in the BCL2 family (Vogler, 2012). Previous studies confirm their association with chemotherapy resistance (Eisele et al., 2007; Yajima et al., 2009; Lane et al., 2015; Sagara et al., 2016; Lin et al., 2020). Besides, both CCL18 and BCL2A1 were significantly related to the occurrence and development of cancer (Yu et al., 2019; Korbecki et al., 2020). This study confirmed their prediction of immunotherapy sensitivity, and we looked forward to more wet experiments to verify it in the future.

The advantages of this paper are as follows: in this study, the relationship between cisplatin chemotherapy and immunotherapy was explored in detail by bioinformatics methods and verified *in vitro* by cell experiment and clinical specimens. The disadvantages of this paper were as follows: for CC patients, we proposed cisplatin chemotherapy combined with immunotherapy for the first time, which urgently needs more *in vitro* and *in vivo* research, including animal experiments.

## Conclusion

COAD patients who were sensitive to cisplatin chemotherapy might also benefit more from immunotherapy. CCL18 and BCL2A1 could simultaneously predict the sensitivity of COAD patients to chemotherapy and immunotherapy. High_CCL18_High_BCL2A1 patients were suitable for combination therapy (chemotherapy and immunotherapy). CCL18, BCL2A1, TOLL-like receptor signaling pathway, and hsa-miR-137 might be novel potential chemotherapy and immunotherapy targets.

## Data Availability

The datasets presented in this study can be found in online repositories. The names of the repository/repositories and accession number(s) can be found in the article/Supplementary Material.
