# Supplementary material for: BCL2A1 and CCL18 Are Predictive Biomarkers of Cisplatin Chemotherapy and Immunotherapy in Colon Cancer Patients
Source: Front Cell Dev Biol. 2022 Feb 21;9:799278. doi: 10.3389/fcell.2021.799278 (PMC8898943; doi:10.3389/fcell.2021.799278)
Supplement: Supplementary file 1 [file Table1.DOCX]

**Table S1.** Composition gene of 10 immune-related signatures.

| Terms | Genes | | | | | | | | | |
| --- | --- | --- | --- | --- | --- | --- | --- | --- | --- | --- |
| PD-L1 Response | IDO2 | CD274 | IDO1 | CTLA4 | LAG3 | TIGIT | TNFSF9 | CD80 | CD70 |  |
| Type I IFN Reponse | DDX4 | IFIT1 | IFIT3 | IRF7 | ISG20 | MX1 | MX2 | RSAD2 | TNFSF10 |  |
| Type II IFN Reponse | GPR146 | SELP | AHR |  |  |  |  |  |  |  |
| Check-point | IDO1 | LAG3 | HAVCR2 | IDO2 | CD274 | PDCD1 | CTLA4 | TIGIT |  |  |
| HLA | HLA-E | HLA-DPB2 | HLA-C | HLA-J | HLA-DQB1 | HLA-DQB2 | HLA-DQA1 | HLA-DQA2 | HLA-A | HLA-DMA |
|  | HLA-DOB | HLA-DRB1 | HLA-H | HLA-B | HLA-DRB5 | HLA-DPB1 | HLA-DRA | HLA-DRB6 | HLA-L | HLA-F |
|  | HLA-G | HLA-DMB | HLA-DPA1 |  |  |  |  |  |  |  |
| MHC class I | B2M | HLA-A | TAP1 |  |  |  |  |  |  |  |
| Parainflammation | CXCL10 | PLAT | CCND1 | LGMN | PLAUR | AIM2 | MMP7 | ICAM1 | MX2 | CXCL9 |
|  | ANXA1 | TLR2 | PLA2G2D | ITGA2 | MX1 | CD276 | TIRAP | IL33 | PTGES | TNFRSF12A |
|  | SCARB1 | CD14 | BLNK | IFIT3 | RETNLB | IFIT2 | ISG15 | OAS2 | REL | CD44 |
|  | RRPAG | BST2 | OAS1 | NOX1 | PLA2G2A | IFIT1 | IFITM3 | IL1RN |  |  |
| Inflammation-promoting | CCL5 | CD19 | CD8B | CXCL10 | CXCL13 | CXCL9 | GNLY | GZMB | IFNG | IL12A |
|  | IL12B | IRF1 | PRF1 | STAR1 | TBX21 |  |  |  |  |  |
| T cell co-stimulation | CD2 | CD226 | CD27 | CD28 | CD40LG | ICOS | SLAMF1 | TNFRSF18 | TNFRSF25 | TNFRSF4 |
|  | TNFRSF8 | TNFRSF9 | TNFSF14 |  |  |  |  |  |  |  |
| T cell co- inhibition | BTLA | VSIR | CD160 | CD244 | CD274 | CTLA4 | HAVCR2 | LAG3 | LAIR1 | TIGIT |

­­­
