# Supplementary material for: BCL2A1 and CCL18 Are Predictive Biomarkers of Cisplatin Chemotherapy and Immunotherapy in Colon Cancer Patients
Source: Front Cell Dev Biol. 2022 Feb 21;9:799278. doi: 10.3389/fcell.2021.799278 (PMC8898943; doi:10.3389/fcell.2021.799278)
Supplement: Supplementary file 2 [file Table2.DOCX]

**Table S2**. The 232 autophagy-related genes (ARG) included in this study were as follows:

| Autophagy-related genes (ARGs) | | | | | | | |
| --- | --- | --- | --- | --- | --- | --- | --- |
| AMBRA1 | APOL1 | ARNT | ARSA | ARSB | ATF4 | ATF6 | ATG10 |
| ATG12 | ATG16L1 | ATG16L2 | ATG2A | ATG2B | ATG3 | ATG4A | ATG4B |
| ATG4C | ATG4D | ATG5 | ATG7 | ATG9A | ATG9B | ATIC | BAG1 |
| BAG3 | BAK1 | BAX | BCL2 | BCL2L1 | BECN1 | BID | BIRC5 |
| BIRC6 | BNIP1 | BNIP3 | BNIP3L | C12orf44 | C17orf88 | CALCOCO2 | CAMKK2 |
| CANX | CAPN1 | CAPN10 | CAPN2 | CAPNS1 | CASP1 | CASP3 | CASP4 |
| CASP8 | CCL2 | CCR2 | CD46 | CDKN1A | CDKN1B | CDKN2A | CFLAR |
| CHMP2B | CHMP4B | CLN3 | CTSB | CTSD | CTSL1 | CX3CL1 | CXCR4 |
| DAPK1 | DAPK2 | DDIT3 | DIRAS3 | DLC1 | DNAJB1 | DNAJB9 | DRAM1 |
| EDEM1 | EEF2 | EEF2K | EGFR | EIF2AK2 | EIF2AK3 | EIF2S1 | EIF4EBP1 |
| EIF4G1 | ERBB2 | ERN1 | ERO1L | FADD | FAM48A | FAS | FKBP1A |
| FKBP1B | FOS | FOXO1 | FOXO3 | GAA | GABARAP | GABARAPL1 | GABARAPL2 |
| GAPDH | GNAI3 | GNB2L1 | GOPC | GRID1 | GRID2 | HDAC1 | HDAC6 |
| HGS | HIF1A | HSP90AB1 | HSPA5 | HSPA8 | HSPB8 | IFNG | IKBKB |
| IKBKE | IL24 | IRGM | ITGA3 | ITGA6 | ITGB1 | ITGB4 | ITPR1 |
| GAA | GABARAP | GABARAPL1 | GABARAPL2 | GAPDH | GNAI3 | GNB2L1 | GOPC |
| GRID1 | GRID2 | KIAA0226 | KIAA0652 | KIAA0831 | KIF5B | KLHL24 | LAMP1 |
| LAMP2 | MAP1LC3A | MAP1LC3B | MAP1LC3C | MAP2K7 | MAPK1 | MAPK3 | MAPK8 |
| MAPK8IP1 | MAPK9 | MBTPS2 | MLST8 | MTMR14 | MTOR | MYC | NAF1 |
| NAMPT | NBR1 | NCKAP1 | NFE2L2 | NFKB1 | NKX2-3 | NLRC4 | NPC1 |
| NRG1 | NRG2 | NRG3 | P4HB | PARK2 | PARP1 | PEA15 | PELP1 |
| PEX14 | PEX3 | PIK3C3 | PIK3R4 | PINK1 | PPP1R15A | PRKAB1 | PRKAR1A |
| PRKCD | PRKCQ | PTEN | PTK6 | RAB11A | RAB1A | RAB24 | RAB33B |
| RAB5A | RAB7A | RAC1 | RAF1 | RB1 | RB1CC1 | RELA | RGS19 |
| RHEB | RPS6KB1 | RPTOR | SAR1A | SERPINA1 | SESN2 | SH3GLB1 | SIRT1 |
| SIRT2 | SPHK1 | SPNS1 | SQSTM1 | ST13 | STK11 | TBK1 | TM9SF1 |
| TMEM49 | TMEM74 | TNFSF10 | TP53 | TP53INP2 | TP63 | TP73 | TSC1 |
| TSC2 | TUSC1 | ULK1 | ULK2 | ULK3 | USP10 | UVRAG | VAMP3 |
| VAMP7 | VEGFA | WDFY3 | WDR45 | WDR45L | WIPI1 | WIPI2 | ZFYVE1 |
