# Supplementary material for: BCL2A1 and CCL18 Are Predictive Biomarkers of Cisplatin Chemotherapy and Immunotherapy in Colon Cancer Patients
Source: Front Cell Dev Biol. 2022 Feb 21;9:799278. doi: 10.3389/fcell.2021.799278 (PMC8898943; doi:10.3389/fcell.2021.799278)
Supplement: Supplementary file 3 [file Table3.DOCX]

**Table S3**. The 259 ferroptosis -related genes included in this study were as follows:

| ferroptosis -related genes | | | | | | | | |
| --- | --- | --- | --- | --- | --- | --- | --- | --- |
| RPL8 | IREB2 | ATP5MC3 | CS | EMC2 | ACSF2 | NOX1 | CYBB | NOX3 |
| NOX4 | NOX5 | DUOX1 | DUOX2 | G6PD | PGD | VDAC2 | PIK3CA | FLT3 |
| SCP2 | TP53 | ACSL4 | LPCAT3 | NRAS | KRAS | HRAS | TF | TFRC |
| TFR2 | SLC38A1 | SLC1A5 | GLS2 | GOT1 | CARS1 | ALOX5 | KEAP1 | HMOX1 |
| ATG5 | ATG7 | NCOA4 | ALOX12 | ALOX12B | ALOX15 | ALOX15B | ALOXE3 | PHKG2 |
| ACO1 | G6PDX | ULK1 | ATG3 | ATG4D | BECN1 | MAP1LC3A | GABARAPL2 | GABARAPL1 |
| ATG16L1 | WIPI1 | WIPI2 | SNX4 | ATG13 | ULK2 | SAT1 | EGFR | MAPK3 |
| MAPK1 | BID | ZEB1 | DPP4 | CDKN2A | PEBP1 | SOCS1 | CDO1 | MYB |
| MAPK8 | MAPK9 | CHAC1 | MAPK14 | LINC00472 | PRKAA2 | PRKAA1 | ELAVL1 | BAP1 |
| ABCC1 | MIR6852 | ACVR1B | TGFBR1 | EPAS1 | HILPDA | HIF1A | IFNG | ANO6 |
| LPIN1 | HMGB1 | TNFAIP3 | TLR4 | ATF3 | ATM | YY1AP1 | EGLN2 | MIOX |
| TAZ | MTDH | IDH1 | SIRT1 | FBXW7 | PANX1 | DNAJB6 | BACH1 | LONP1 |
| PTGS2 | DUSP1 | NOS2 | NCF2 | MT3 | UBC | ALB | TXNRD1 | SRXN1 |
| GPX2 | BNIP3 | OXSR1 | SELENOS | ANGPTL7 | SLC7A11 | DDIT4 | LOC284561 | ASNS |
| TSC22D3 | DDIT3 | JDP2 | SESN2 | SLC1A4 | PCK2 | TXNIP | VLDLR | GPT2 |
| PSAT1 | LURAP1L | SLC7A5 | HERPUD1 | XBP1 | SLC3A2 | CBS | ATF4 | ZNF419 |
| KLHL24 | TRIB3 | ZFP69B | ATP6V1G2 | VEGFA | GDF15 | TUBE1 | ARRDC3 | CEBPG |
| SNORA16A | RGS4 | BLOC1S5-TXNDC5 | LOC390705 | EIF2S1 | KIM-1 | IL6 | CXCL2 | RELA |
| HSD17B11 | AGPAT3 | SETD1B | FTL | MAFG | IL33 | FTH1 | SLC40A1 | GPX4 |
| HAMP | HSPB1 | NFE2L2 | STEAP3 | DRD5 | DRD4 | MAP3K5 | SLC2A1 | SLC2A3 |
| SLC2A6 | SLC2A8 | SLC2A12 | GLUT13 | SLC2A14 | EIF2AK4 | TFAP2C | SP1 | HBA1 |
| NNMT | PLIN4 | HIC1 | STMN1 | RRM2 | CAPG | HNF4A | NGB | YWHAE |
| GABPB1 | AURKA | MIR4715 | RIPK1 | PRDX1 | MIR30B | AKR1C1 | AKR1C2 | AKR1C3 |
| RB1 | HSF1 | GCLC | SQSTM1 | NQO1 | MUC1 | MT1G | CISD1 | FANCD2 |
| FTMT | HSPA5 | HELLS | SCD | FADS2 | SRC | STAT3 | PML | MTOR |
| NFS1 | TP63 | CDKN1A | MIR137 | ENPP2 | FH | CISD2 | MIR9-1 | MIR9-2 |
| MIR9-3 | ISCU | ACSL3 | OTUB1 | CD44 | LINC00336 | BRD4 | PRDX6 | MIR17 |
| NF2 | ARNTL | JUN | CA9 | TMBIM4 | PLIN2 | MIR212 | Fer1HCH | AIFM2 |
| LAMP2 | ZFP36 | PROM2 | CHMP5 | CHMP6 | CAV1 | GCH1 |  |  |
